# Acetaminophen Levels Found in Recycled Wastewater Alter Soil Microbial Community Structure and Functional Diversity

**DOI:** 10.1007/s00248-022-02022-8

**Published:** 2022-05-04

**Authors:** Nathan K. McLain, Melissa Y. Gomez, Emma W. Gachomo

**Affiliations:** Department of Microbiology and Plant Pathology, University of California, Riverside, Riverside, CA USA

**Keywords:** Contaminants of emerging concern, Treated wastewater, Soil bacterial community, Soil microbiome

## Abstract

The practice of using recycled wastewater (RWW) has been successfully adopted to address the growing demand for clean water. However, chemicals of emerging concern (CECs) including pharmaceutical products remain in the RWW even after additional cleaning. When RWW is used to irrigate crops or landscapes, these chemicals can enter these and adjacent environments. Unfortunately, the overall composition and concentrations of CECs found in different RWW sources vary, and even the same source can vary over time. Therefore, we selected one compound that is found frequently and in high concentrations in many RWW sources, acetaminophen (APAP), to use for our study. Using greenhouse grown eggplants treated with APAP concentrations within the ranges found in RWW effluents, we investigated the short-term impacts of APAP on the soil bacterial population under agricultural settings. Using Illumina sequencing-based approaches, we showed that APAP has the potential to cause shifts in the microbial community most likely by positively selecting for bacteria that are capable of metabolizing the breakdown products of APAP such as glycosides and carboxylic acids. Community-level physiological profiles of carbon metabolism were evaluated using Biolog EcoPlate as a proxy for community functions. The Biolog plates indicated that the metabolism of amines, amino acids, carbohydrates, carboxylic acids, and polymers was significantly higher in the presence of APAP. Abundance of microorganisms of importance to plant health and productivity was altered by APAP. Our results indicate that the soil microbial community and functions could be altered by APAP at concentrations found in RWW. Our findings contribute to the knowledge base needed to guide policies regulating RWW reuse in agriculture and also highlight the need to further investigate the effects of CECs found in RWW on soil microbiomes.

**Supplementary Information:**

The online version contains supplementary material available at 10.1007/s00248-022-02022-8.

## Introduction

Potable water supplies are becoming scarce with the increasing world population. Changing climate factors such as rising temperatures and altered precipitation patterns limit the regeneration of these supplies [1–3]. Conservation can only stretch water supplies so far and may not be enough to address the growing demands for clean water [2]. Alternative ways to generate usable water, such as recycling wastewater, are essential to help meet the rising demand [2, 4]. Efforts to solve water shortage problems by importing water can impact non-arid regions and can cause the environment to suffer through reduction of habitat area and water availability for the biota [5]. In addition, importing water can be costly and it is not an ideal long-term solution [6, 7]. Therefore, alternative methods for generating potable water are becoming a necessity to meet rising water demands [3, 8].

Use of recycled wastewater (RWW) to supplement potable water supplies has been very successful [7–9] in arid regions, such as southern California. RWW is primarily used for agriculture and landscape irrigation [8–11], allowing farmers in arid regions to maintain high agricultural outputs with less dependence on the potable water supply or having to increase water withdrawal from natural aquifers [7, 11]. Using RWW has been so successful that many water districts in California are planning on increasing their capacity for capturing and treating larger volumes of their wastewater [12]. Despite the large conservation success of RWW, this water may pose risks to natural and agricultural environments. The wastewater treatment process is efficient at removing potentially disease-causing biological contaminants, but is less effective at removing chemical contaminants [13–16]. These contaminants include pharmaceuticals, personal care products, detergents, and nanoparticles that are collectively referred to as chemicals of emerging concern (CECs) [14, 16, 17]. Processing of wastewater can reduce the levels of CECs by major proportions depending upon the RWW plant (for example: 74%, 71%, 67%, 91%, 99% for sulfamethoxazole, tetracycline, gemfibrozil, ibuprofen, and APAP respectively) [14, 16, 17]. It was originally believed that the final CEC concentrations in RWW effluent (typically in the μg to mg/L range) were too low to be biologically relevant [18–20]. However, recent evidence suggests that the concentrations of CECs in RWW can impact microorganisms, insects, and plants [21–26]. CECs accumulate in soils irrigated with RWW [14], and they are taken up by plants inevitably accumulating in their tissues [18, 19, 27]. Therefore, CECs pose a risk in the agricultural settings where RWW is primarily used, and RWW should be evaluated extensively to manage or reduce any potential hazards.

RWW sources vary in the concentration and composition of CECs temporally and spatially, which may be related to the sources and human activities that generate the wastewater [28]. Additionally, it is more than likely that the different CECs will interact with each other and affect the behavior of different CECs that are present, possibly ameliorating or intensifying their effects. For example, gemfibrozil can increase the potency of the antifungal compound fluconazole [29], sulfamethoxazole can increase the antimicrobial effects of rifampicin [30], and APAP can induce β-lactamase activity and decrease the susceptibility of bacteria to certain antibiotics [20]. Aspirin and ciprofloxacin can have antagonistic drug interactions, reducing their potency [31]. These combinations of factors make it difficult to distinguish the impacts of individual CECs in RWW on the plant associated microbial communities. Therefore, we decided to examine the effects of one of the most prevalent CEC on soil microbes. APAP is consistently found in RWW effluent in many regions and at higher concentrations than other CECs [13, 16, 28, 32, 33]. At the upper ranges, APAP has been found to reach max concentrations of 24.53 to 112.78 ug/L [34, 35]. Average concentrations of APAP in RWW effluent have been found between 0.0081 ug/L [14] to 11.73 ug/L [35]. APAP has also been observed to accumulate in soils from 10 to 4860% above the concentration found in the applied effluent [14].

APAP can act as an anthropogenic factor and disrupt microbial functions essential to plant health such as nitrogen cycling [22]. Soil microbiomes that are altered by anthropogenic factors can exhibit the loss or reduction of key functions, such as nutrient cycling [36–38]. While most studies to date on the hazards of CECs in the soil have focused on accumulation, transformation, or effects on soil fertility, [3, 14, 34, 39], few if any have investigated their impacts on plant–microbe interactions. In this study, we investigated how APAP can alter the soil microbiome and consequently impact plant health which is correlated to productivity [40–42]. Since CECs accumulate in soils irrigated with RWW, they can alter the plant associated soil microbiome [22]. The addition of CECs into a given soil environment has the potential to select for a specific group of organisms, possibly ones that can benefit directly from the compound [43]. Since high usage of APAP is likely to continue, and it has been found to impact soil microorganisms and their functions, we decided to evaluate the short-term (3 and 7 weeks post application) impacts directly on the soil microbial community of an important agricultural crop. We hypothesized that APAP at levels found in RWW will alter the soil bacterial community structure and function within a single growing season.

## Materials and Methods

### Eggplant Cultivation and Soil Collection

*Solanum melongena* (eggplants, variety Patio Baby) were cultivated as described in supplementary material and methods (SI-1) and our previous study [26]. Treatments were applied by irrigating with water containing 10 μg/L or 5 μg/L of APAP (APAP-10 and APAP-5 respectively), and control plants (no CEC) with tap water [44, 45]. Tap water sources are independent to the RWW system and previous observations have indicated that tap water contains a negligible amount of CECs [46]. Given that the composition of RWW is very variable [14, 16, 17] and that RWW contains a plethora of compounds that may impact the plants or soil microbes directly [14, 16, 17], we decided to dilute APAP in tap water to reduce the number of factors that could contribute to the results obtained. Soil samples were collected before treatments (T0 time point), 3 and 7 weeks after beginning of treatments (T1 and T2 respectively). Push cores of 1-cm diameter and 3 cm deep of soil were collected at least 3 cm from the eggplant stem and 3 cm from the wall of the pot containing the soil. Please see the Supplementary Methods for more details.

### DNA Extractions and Illumina Sequencing Library Preparation

Total environmental DNA was extracted from 0.25 g of soil samples described above using the DNeasy Powersoil kit (Qiagen, Valencia, CA, USA), following manufacturer instructions, except 50 µL of solution C6 was used. DNA quality was checked using an Implen NanoPhotometer (Implen, Westlake Village, CA, USA). Amplicon libraries of the bacterial 16S rRNA gene were generated from the extracted DNA to characterize the bacterial community. A two-step PCR dual indexing inline barcoding procedure and primers were used to generate amplicons for Illumina sequencing [23, 47, 48]. Phusion high-fidelity PCR master mix with HF buffer (Thermo Scientific) and 0.2-µM primers were used as PCR reagents with 1 µL of extracted DNA for the template. PCRs were carried out on the BioRad T100 thermal cycler as described by Kembel and colleagues [23, 48] except we used 56.5 annealing temperature, 24 cycles, and final elongation time of 5 min. PCRs were screened for quality and fragment size using gel electrophoresis with a 1% agarose gel. Amplicons from successful PCRs were purified using the Agencourt AMPure xp beads protocol (Beckman Coulter, Brea, CA, USA), except that SPRI beads (Beckman Coulter, Brea, CA, USA) were used and all ethanol washes were done using 80% ethanol. Cleaned DNA products were used as a template in a second PCR under similar conditions as described above except 0.3 µM HPLC-purified PCR2F and PCR2R primers were used [23, 48] and 7 cycles were used with an annealing temperature of 65 °C. PCRs were screened as described for the initial PCR. DNA concentrations were measured using the nanodrop spectrophotometer, and amplicons were pooled in equal molar concentrations of 5 nM for sequencing. The samples were submitted to the UCR genomics core facility where library quality was assessed using a 2100 Bioanalyzer (Agilent) and the libraries were sequenced using a MiSeq sequencer (Illumina) and Miseq Reagent kit version 3 (Illumina) with 2 × 150 cycles. The Raw sequences were submitted to NCBI and are under the accession numbers PRJNA808107.

### Data Analysis—Processing and Quality filtering

The forward and reverse Illumina sequencing reads were joined together and quality filtered using default settings in QIIME1 [49]. Joined sequences were demultiplexed using their unique barcode pairs in QIIME1. Demultiplexed samples were uploaded into QIIME2 with their associated quality scores [50]. Sequences were quality filtered further using the deblur method in QIIME2 [50–52]. Samples that contained less than 9000 sequences were removed. The number of sequences per sample were rarefied down to match the sample with the lowest amount, 10,300 sequences [50]. Deblur classified these sequences into amplicon sequence variants (ASVs) that were taxonomically identified to the lowest possible level by matching to the Greengenes database (v 13.8) using QIIME2 default parameters [53]. Negative controls were sequenced in parallel, any ASVs detected were filtered out from the data using QIIME2 before downstream analyses. Community α-diversity was measured using the Shannon-Wiener index in QIIME2 and statistically compared using the best fitting generalized linear model (GLiM) (normal distribution and identity link function) as determined by the model with the lowest Akaike’s Information Criterion (AIC) in SPSS (IBM, V. 27.0). Box plots of α-diversity metrics were generated in QIIME2. Community differences among all time points (β-diversity) were evaluated using PERMANOVA [54, 55] on Bray–Curtis distance matrices in QIIME2 [56, 57]. Boxplots of the β-diversity were plotted in QIIME2. Community data from QIIME2 was used in Paleontological Statistics (PAST) [58] to generate PCA graphs showing the taxa that contributed to the most differences among communities. The group significance test in QIIME1, which uses pairwise Kruskal–Wallis tests, was used to statistically compare the abundance of ASVs [49]. Taxa were considered to be significantly different in relative abundance if *P* < 0.05, with an FDR value lower than 0.2. A conservative FDR value of less than 0.2, as described by Efron [59], was used in order to obtain a more inclusive set of microbes that are potentially impacted by APAP so that more bacterial taxa could be considered for additional study. A similar logic was used by Go et al. [60] to screen for candidate metabolites, and the study Kong et al. [61] used FDR < 0.2 to determine which microbes were significantly differentially abundant in the oral and gut microbiome of humans. Community data generated in QIIME2 was imported into PICRUSt2 [62] to predict the potential bacterial metagenome present in the soil communities. The data was normalized by copy number and predictions were based on the Kyoto Encyclopedia of Genes and Genomes (KEGG orthologs) database. STAMP [63] was used to do initial ANOVAs on each predicted gene to screen for ones that were differentially abundant among all treatments. Genes that were found to be significantly differentially abundant (*P* < 0.05) with a high effect size (measured as eta-squared (*ƞ*^2^)), *ƞ*^2^ > 0.40, were kept for additional pairwise analyses described below to ensure that the differences were biologically relevant [64]. Welch’s *t*-test, in STAMP, was used for pairwise comparisons among all treatments for genes that passed this screening.

### Evaluating Changes in Microbial Functional Diversity

In order to verify PICRUSt2 predictions and determine changes in functional diversity, the utilization of different carbon sources for microbes in APAP-10-treated and untreated soils was evaluated using the Biolog EcoPlate [65, 66]. The Biolog EcoPlate contains 31 ecologically relevant carbon sources and water (control) in triplicates within a 96 well plate (Supplementary Table 1). The same soil samples collected at 7 weeks after treatment with APAP-10 (T2), described above, were used in the Biolog procedure described by Liu et al. [65] with a few exceptions. To make soil suspensions, 1 g soil was added to 10 mL of dH_2_O, shaken at room temperature, added to the Biolog plates that were incubated for 6 days at 25 °C, and the absorbance at 590 nm was read at 12, 24, 48, 72, 96, and 120 h post inoculation (hpi) using a Promega GloMax-Multi Detection System.

The absorbance of each well was standardized by subtracting the absorbance for the water control. Average well color development (AWCD) was used as a measure utilization of the carbon source in each well by the microbial community. The formula used to calculate AWCD was as follows:

$$\mathrm{AWCD}=\sum \frac{(\mathrm{Optical Density in Carbon Source Well}-\mathrm{Optical Density of Control})}{31}$$

A 3-way best fitting generalized linear model (GLiM) (gamma distribution with log link function) as determined by the model with the lowest AIC was used to determine the interaction effect of APAP treatment and their respective impacts on AWCD. One-way GLiMs were used to compare the effects of soil treatment among time points. GLiM, post hoc pairwise comparisons were done using the least significant difference (LSD) to evaluate treatment effects within each time point. Biolog plate and community data from QIIME2 were imported into PAST to conduct canonical correspondence analysis (CCA).

Hydrolysis of fluorescein diacetate (FDA) was used as a proxy to measure microbial activity in soils treated with APAP-10 and without APAP. Soil similar to that used to grow eggplants as described above was irrigated with APAP-10 in the greenhouse for 3 weeks, with no plants grown in it. Each treatment was replicated 4 times. The FDA assay and standard curve were carried out as described in [67], with the exception that 6.0 g of wet weight soil were incubated for 15 h at 30 ℃. For the standard curve, 50 mL acetone solutions containing 0 to 800 ug of FDA, in increments of 200 ug, were measured using spectrophotometry.

## Results

### Bacterial Community

**Fig. 1:**
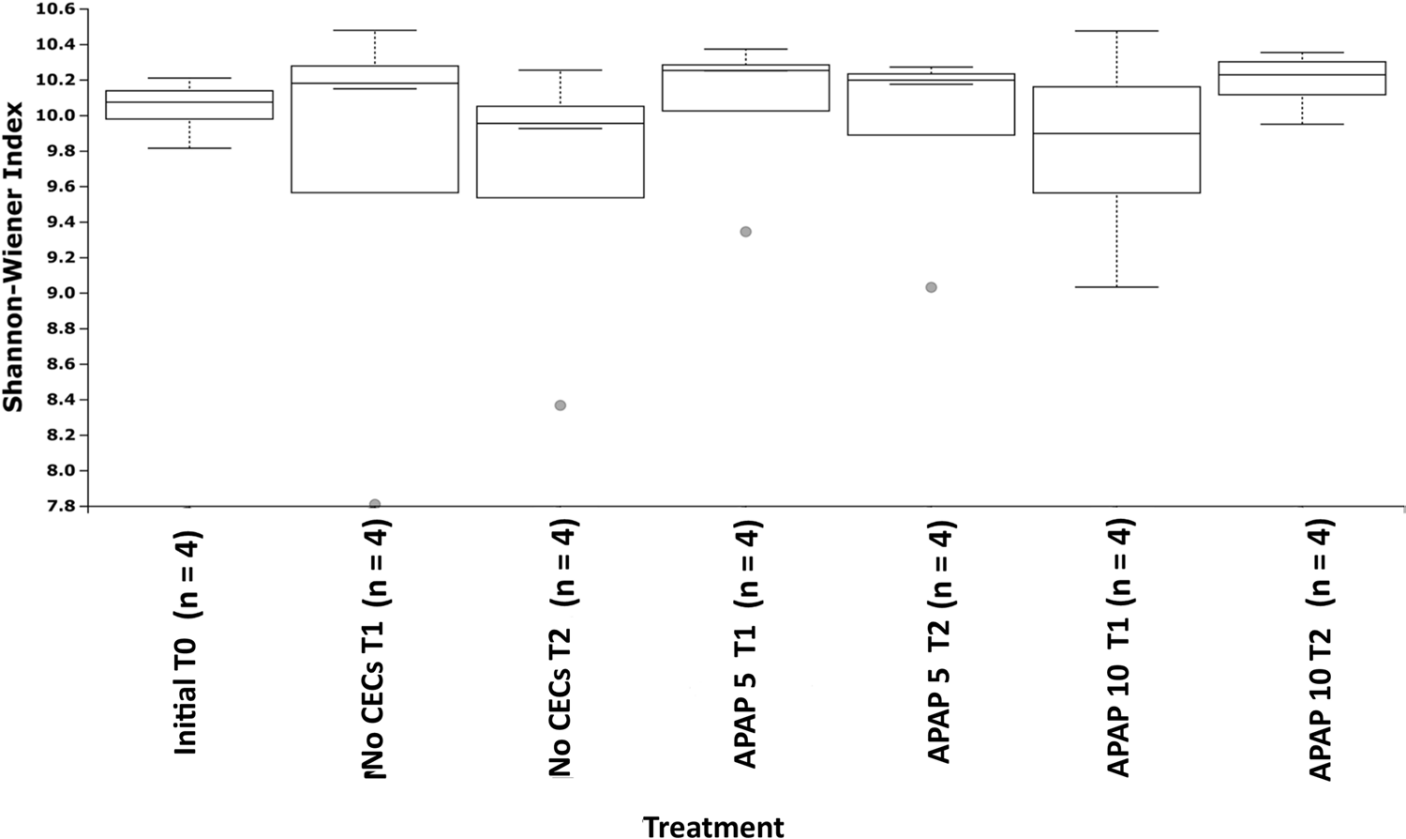
Box plots comparing the Shannon-Wiener index of samples treated with 10 μg/L (APAP 10) or 5 μg/L of acetaminophen (APAP 5) and the untreated control (no CEC) collected at the beginning of the experiment, 3 and 7 weeks after start of treatment (T0, T1, and T2 respectively)

Illumina sequencing data indicated that APAP did not have an effect on community ⍺-diversity. APAP-10 at T2 had the highest Shannon–Wiener index value of 10.18, while the no CEC treatment at T2 had the lowest at 9.63 (Fig. 1). The initial diversity present in the soil community at T0 was 10.04. These differences in diversity were not impactful, as no significant interactions nor differences were detected among treatments and time points (2-way GLiM:${x}^{2}_{2}=0.744$, $P=0.679$; ${x}^{2}_{2}=1.874$, $P=0.392$; ${x}^{2}_{2}=0.078$, $P=0.780$; respectively).

**Fig. 2:**
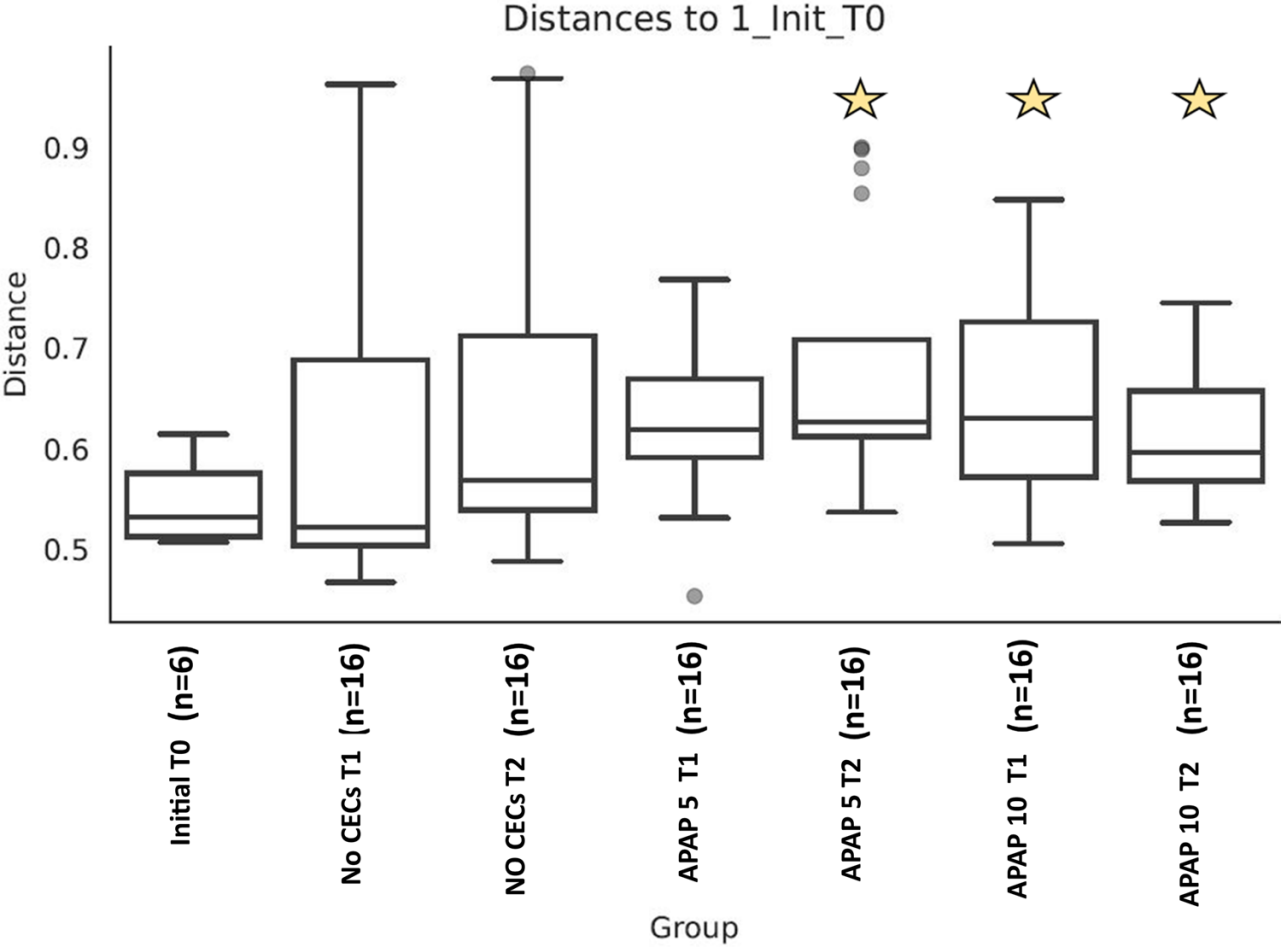
Box plot of Bray Curtis distance samples untreated and treated with 10 μg/L or 5 μg/L of acetaminophen (APAP 10 or APAP 5 respectively) and the untreated control (no CEC), and collected at the beginning of the experiment, 3 and 7 weeks after start of treatment (T0, T1, and T2 respectively). Star denotes samples that were significantly different from diversity values compared to the initial, T0, samples. Pairwise Permanova all *P*’s < 0.05

**Fig. 3:**
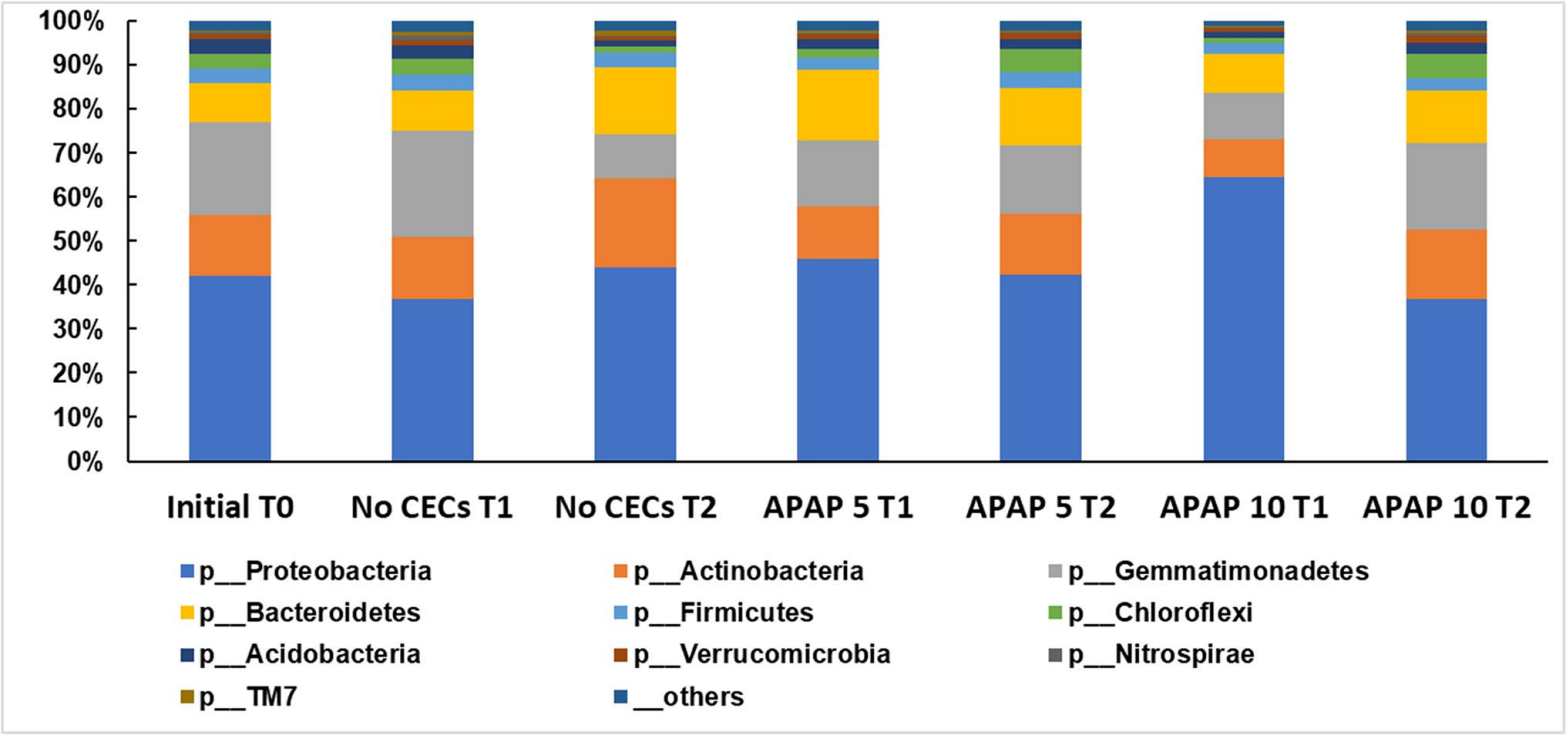
The relative abundance of bacteria in soil samples treated with 10 μg/L or 5 μg/L of acetaminophen (APAP 10 or APAP 5 respectively) and the untreated control (no CEC), and collected at the beginning of the experiment, 3 and 7 weeks after start of treatment (T0, T1, and T2 respectively). Relative abundance of the bacteria was determined at the phylum level only. *P* = phylum

However, the community structure was altered by the addition of APAP. The initial overall PERMANOVA comparison did not detect significant differences among treatments (PERMANOVA pseudo-*F* = 1.85, pseudo-*P* = 0.149), while pairwise analyses did. APAP-10-T1 soil community structure was significantly different from the T0 soil community (PERMANOVA pseudo-*F* = 1.743, pseudo-*P* = 0.047) (Fig. 2). At T2, the soil community structures treated with APAP-10 and APAP-5 were significantly different from the T0 soil community (PERMANOVA pseudo-*F* = 2.100, pseudo-*P* = 0.026; pseudo-*F* = 1.749, pseudo-*P* = 0.016; respectively). At T1 and T2, the untreated soil community structure was not significantly different from the T0 community (PERMANOVA all *P*’s > 0.05). The relative abundance of different bacterial groups was impacted by the addition of APAP. A total of 748 ASVs were identified among all samples, and 247 of them were found to be significantly differentially abundant between T0 and the APAP-10 T2 communities (QIIME1 group significance Kruiskal-Wallis test; all *P*’s < 0.05, All FDR < 0.17). In all treatments, Proteobacteria were the most abundant in the soil with a relative abundance between 40 and 60% (Fig. 3). The relative abundance of the Chloroflexi phylum more than doubled in any soils treated with APAP, but decreased in the untreated soils between T1 and T2 (Fig. 3). The relative abundance of the Actinobacteria class increased from 6.3% and 8.6% at T1 to 9.8% and 11.0% at T2 for APAP-10 and APAP-5, respectively. This was lower than in the untreated soil that had relative abundance of 9.4% at T1 and 17.8% at T2 (Table 1). Bacteroidetes phylum abundance was lower in APAP-treated soil than untreated soils by time point T2 with only 11.9% and 13.2% relative abundance for APAP-10 and -5 respectively, compared to 15.2% for the untreated soil community. Indicating an inverse relationship between abundance of the Bacteroidetes phylum and APAP concentration. At T2, the Gemmatimonadetes class had higher relative abundance in the APAP-10- and APAP-5-treated soil (8.3% and 6.5%, respectively) compared to the untreated soil (4.4%) (Table 1). The relative abundance of Firmicutes did not change significantly with time or APAP treatment, and remained between 2.3 and 3.6%. However, the relative abundance of Acidobacteria decreased in all treatments compared to the original soil and the largest decrease was observed in the untreated soil (3.3 to 1.3%; T0 to T2 respectively) (Table 1).

**Table 1:** Relative abundance averaged among replicate samples of major taxonomic groups detected in the soil from Illumina sequencing

Taxonomy	Initial T0	No CECs T1	No CECs T2	APAP 5 µg/L T1	APAP 5 µg/L T2	APAP 10 µg/L T1	APAP 10 µg/L T2
p__Proteobacteria;c__Betaproteobacteria	5.43%	4.01%	2.49%	3.48%	3.91%	2.22%	3.79%
p__Proteobacteria;c__Deltaproteobacteria;o__Myxococcales	4.10%	4.10%	4.48%	4.02%	4.46%	2.13%	4.24%
p__Proteobacteria;__others	3.42%	2.95%	2.25%	2.69%	2.70%	12.17%	2.73%
p__Proteobacteria;c__Gammaproteobacteria;o__Xanthomonadales	8.63%	6.52%	6.76%	6.54%	7.38%	0.01%	8.05%
p__Proteobacteria;c__Gammaproteobacteria;o__Pseudomonadales	3.99%	1.34%	5.05%	2.93%	1.17%	0.53%	1.19%
p__Proteobacteria;c__Gammaproteobacteria;__others	2.22%	2.95%	3.06%	5.63%	4.05%	23.29%	2.72%
p__Proteobacteria;c__Alphaproteobacteria;o__Sphingomonadales	5.47%	5.14%	6.78%	5.55%	5.94%	0.32%	5.62%
p__Proteobacteria;c__Alphaproteobacteria;o__Rhizobiales	2.03%	2.99%	4.87%	4.85%	4.17%	2.82%	2.72%
p__Proteobacteria;c__Alphaproteobacteria;__others	6.83%	6.91%	8.17%	10.32%	8.45%	21.15%	5.67%
p__Actinobacteria;c__Actinobacteria	9.34%	9.38%	17.76%	8.58%	9.81%	6.29%	11.05%
p__Actinobacteria;__others	4.59%	4.74%	2.68%	3.28%	4.10%	2.09%	4.95%
p__Gemmatimonadetes;c__Gemmatimonadetes	9.07%	10.83%	4.42%	6.35%	6.51%	4.05%	8.25%
p__Gemmatimonadetes;__others	11.78%	13.18%	5.50%	8.61%	8.99%	6.59%	11.22%
p__Bacteroidetes	8.91%	9.34%	15.17%	16.03%	13.24%	9.05%	11.92%
p__Firmicutes	3.49%	3.57%	3.34%	2.78%	3.46%	2.31%	2.85%
p__Chloroflexi	3.20%	3.55%	1.51%	2.09%	5.46%	1.26%	5.52%
p__Acidobacteria	3.30%	3.08%	1.31%	2.22%	2.09%	1.29%	2.51%
p__Verrucomicrobia	1.20%	1.03%	0.82%	1.06%	1.47%	0.73%	1.76%
p__Nitrospirae	0.67%	1.15%	0.24%	0.43%	0.27%	0.26%	0.41%
p__TM7	0.08%	0.72%	1.16%	0.35%	0.30%	0.29%	0.54%
Others	2.24%	2.52%	2.16%	2.22%	2.07%	1.15%	2.28%

**Fig. 4:**
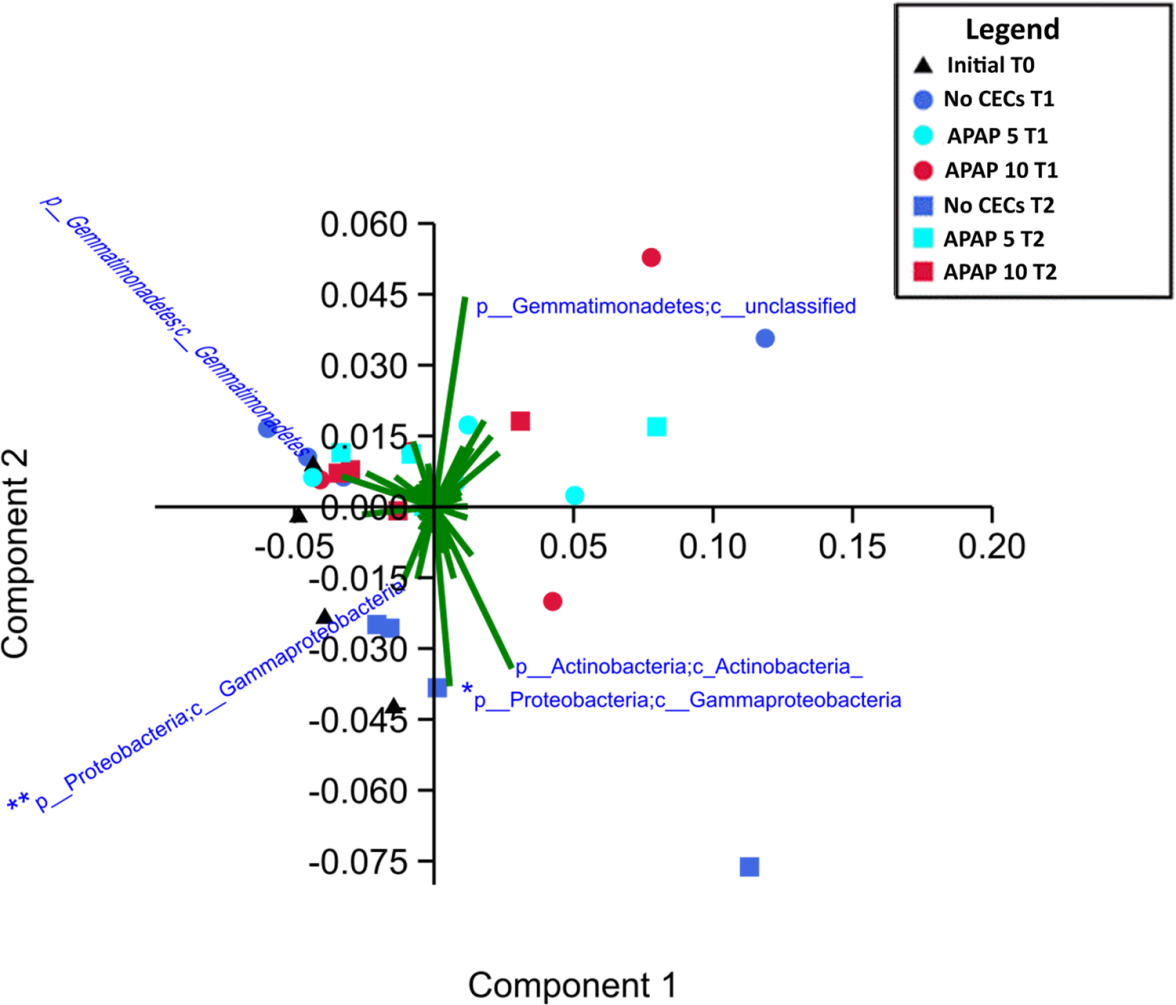
PCA graph of Illumina sequencing data with plotted vectors showing community members that contributed most to the variability in soil communities from soil treated with 10 μg/L or 5 μg/L of acetaminophen (APAP-10 and APAP-5 respectively) and the untreated control (no CEC), and collected at the beginning of the experiment, 3 and 7 weeks after start of treatment (T0, T1, and T2 respectively). * = Gammaproteobacteria class containing the Pseudomonadaceae family, ** = Gammaproteobacteria class containing the Xanthomonadaceae family

The PCA plot of the sequencing data revealed 5 taxonomic groups that had a strong impact on causing community differences among the treatments (Fig. 4). The Actinobacteria class contained numerous lower divisions of microbial taxa, with the majority of their abundance being significantly lower in APAP-10-treated soils than the initial soil T0 (QIIME1 group significance Kruskal–Wallis; all *P*’s < 0.05). There were two distinct groups of microbes in the Gemmatimonadetes phylum that decreased significantly in abundance from T0 to T2 in the APAP-10-treated soils (QIIME1 group significance Kruskal–Wallis; all *P*’s < 0.05, all FDR < 0.13). The Pseudomonadaceae family makes up a large component of the vector representing the Gammaproteobacteria class (denoted with * in Fig. 4), and this family significantly decreased in abundance between T0 and T2 in APAP-10 (QIIME1 group significance Kruskal–Wallis; all *P*’s < 0.05). The decrease in abundance of Xanthomonadaceae family within the Gammaproteobacteria class (denoted with ** in Fig. 4) after APAP-10 treatment was not significant (QIIME1 group significance Kruskal–Wallis; *P* > 0.05, FDR > 0.13; Fig. 4). However, a few individual organisms of agricultural importance in Xanthomonadaceae and Pseudomonadaceae families increased with the addition of APAP, namely *Lysobacter* spp. and *Pseudomonas viridiflava*, respectively, whose relative abundance was 0.064% and 0.26% higher in APAP-10-treated soil compared to the untreated control by T2.

### Metagenome Prediction

**Fig. 5:**
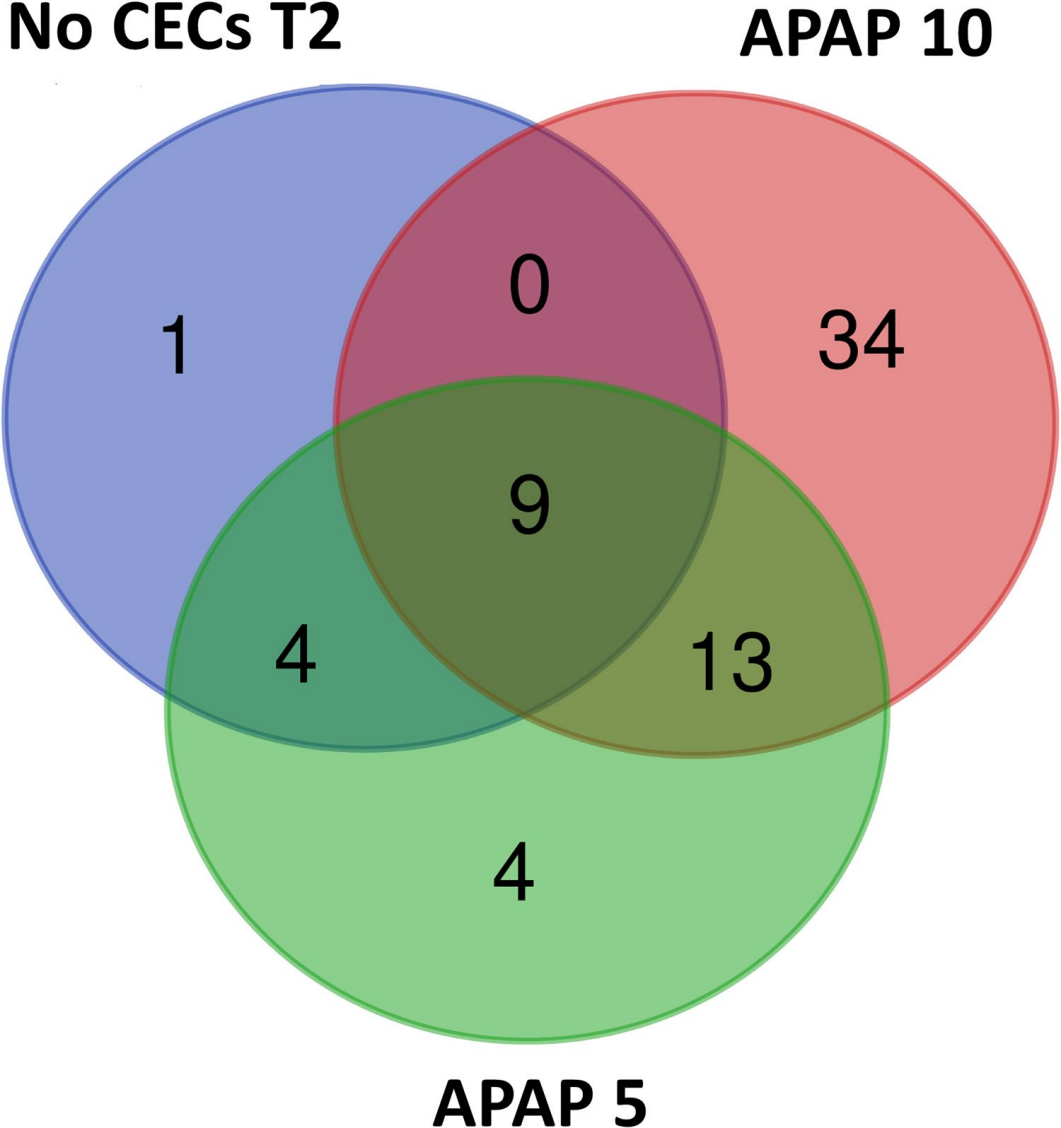
Venn diagram comparing genes predicted to be significantly more abundant in soil communities from soil treated with 10 μg/L or 5 μg/L of acetaminophen (APAP-10 and APAP-5 respectively) and the untreated control (no CEC), and collected at the beginning of the experiment and 7 weeks after start of treatment (T0 and T2 respectively) compared to the initial T0 communities (Welch’s *T* test, all, all *P*’s < 0.05)

Interestingly, the PICRUSt2 metagenome analysis predicted there to be 7393 potentially expressed genes among all soil bacterial communities in this study. The initial ANOVAs to screen for biologically relevant differences in gene abundance among treatments found 521 such genes. According to the Kyoto Encyclopedia of Genes and Genomes (KEGG) database, 202 of them were involved in metabolic pathways [68] (ANOVAs, all *P*’s < 0.05; all ƞ^2^ > 0.40). At T2, APAP-10 had more genes predicted to be significantly differentially abundant than T0, no CEC or APAP-5 treatments (Fig. 5, Supplementary Table 2). APAP-10 T2 had 47 predicted genes that were significantly greater in abundance compared to the initial soil community.

A diverse set of predicted metabolic genes had increased in abundance by T2 compared to T0. The majority were observed in APAP-treated soil communities. APAP-10 T2 had many predicted upregulated genes related to amino acid, carbohydrate, energy, cofactors, and vitamins, terpenoids and polyketides metabolism, and biosynthesis of other secondary metabolites (Supplementary Table 2), but the no CEC soil community had only one metabolic gene predicted to be increased. Additionally, 92% of the predicted genes for the metabolism of terpenoids and polyketides were observed in APAP-treated soil communities and about 70% of them were in the APAP-10 T2 soil community. Overall, APAP-10 T2 had the highest number of predicted genes to increase in abundance which were in more diverse metabolism categories compared to the other treatments (Supplementary Table 2).

### Evaluating Changes in Microbial Functional Diversity

**Fig. 6:**
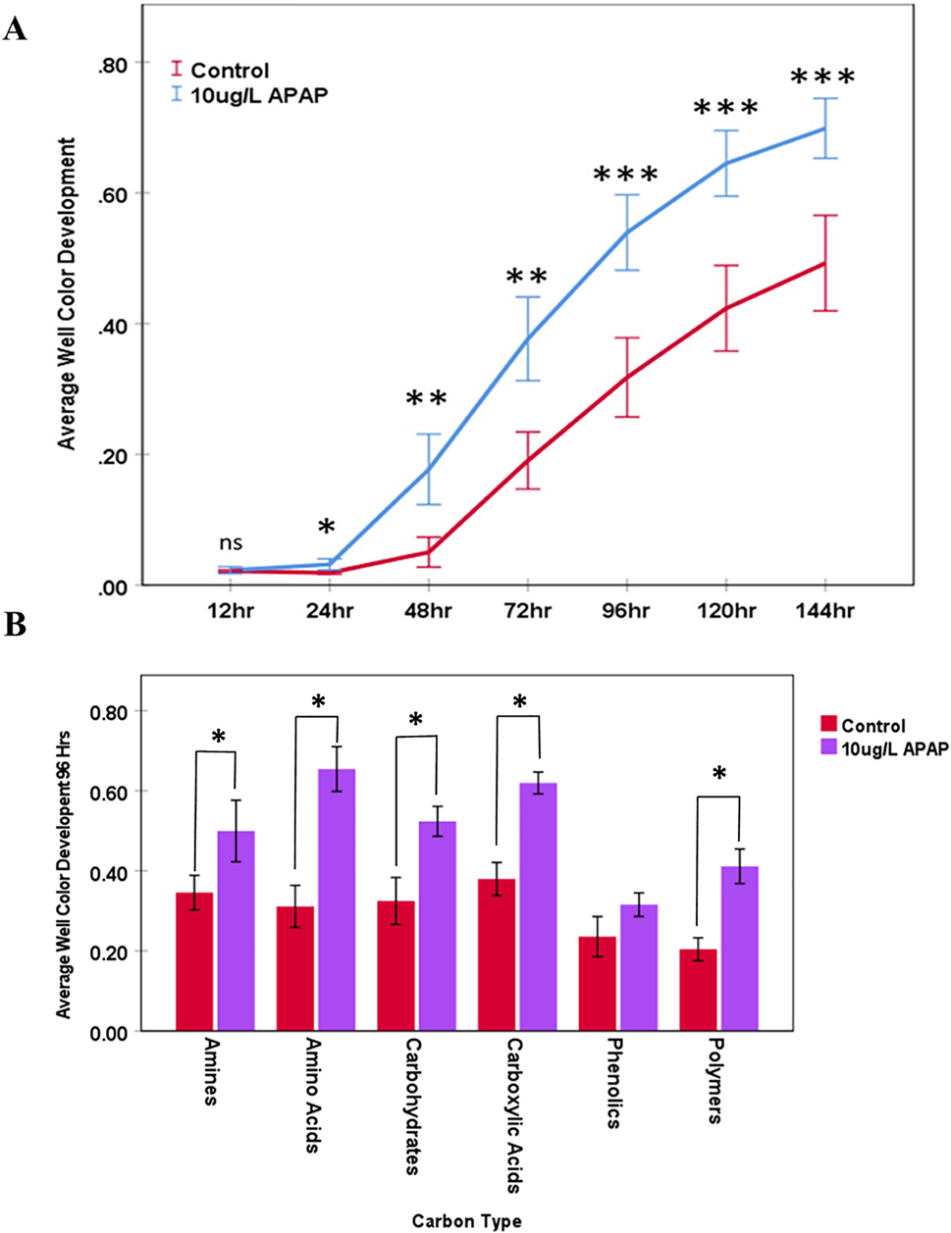
Average Well Color Development in Biolog EcoPlate wells containing samples from soil treated with 10 μg/L of acetaminophen (10 μg/L APAP) and the untreated control collected 7 weeks after start of treatment. **A** Total Average Well Color Development for all carbon sources in treated and control samples over the course of 144 h. **B** Average Well Color Development of each functional carbon group 96 h after incubation. For all graphs, error bars represent the standard error. Lines between treatments represent GLim post hoc LDS comparisons between control and 10 µg/L APAP. Single asterisks represent a *P*-value < 0.05, double asterisks represent a *P*-value < 0.01, and triple asterisks represent a *P*-value < 0.001

The PICRUSt2 analysis of the expected metagenome predicted there to be a higher abundance of metabolic genes in the APAP-10 T2 soil community compared to the other treatments, suggesting there to be higher rates of metabolism in the APAP-10 soil community. Therefore, these predictions were confirmed by evaluating soil community functions using the Biolog Ecoplate assay. The breakdown of various carbon sources directly (measured as the average well color development, AWCD) serves as a proxy to measure soil community activity [66]. Across all the time points, carbon sources, and CEC treatments, carbon utilization (measured as AWCD) was significantly higher in APAP-10-treated soil compared to the control (3 way GLiM: *X*^2^ = 190.327, *P* ≤ 0.001). Carbon utilization was also significantly different among carbon types and timepoints (3 way GLiM: *X*^2^ = 86.067, *P* ≤ 0.001 & *X*^2^ = 3253.563, *P* ≤ 0.001; respectively). A significant 3-way interaction was detected between APAP treatment, carbon type, and time point (3-way GLiM: *X*^2^ = 54.522, *P* = 0.003). Significant 2-way interactions were detected among CEC treatments and carbon type, CEC treatments and timepoints, and carbon type and timepoints (3-way GLiM: *X*^2^ = 40.705, *P* ≤ 0.001; *X*^2^ = 56.559, *P* ≤ 0.001; *X*^2^ = 182.62, *P* ≤ 0.001; respectively). From 24 h post incubation (hpi) to 144 hpi, the AWCD of APAP-10-treated soils was significantly higher than that of untreated soils (one-way GLiM: *X*^2^ = 2544.759, *P* ≤ 0.001; all post hoc LSD comparisons < 0.05). (Fig. 6A). The AWCD of APAP-treated soils were 1.3%,12.7%, 18.7%, 22.2%, 22.2%, and 20.6% higher than untreated soils after 24, 48, 72, 96, 120, and 144 hpi, respectively (Fig. 6A). By 96 hpi, carbon utilization for amines, amino acids, carbohydrates, carboxylic acids, and polymers were significantly higher for APAP-treated soils than untreated soils (One-way GLiM: *X*^2^ = 101.607, *P* ≤ 0.001; all post hoc LSD *P* < 0.05; Fig. 6B).

**Fig. 7:**
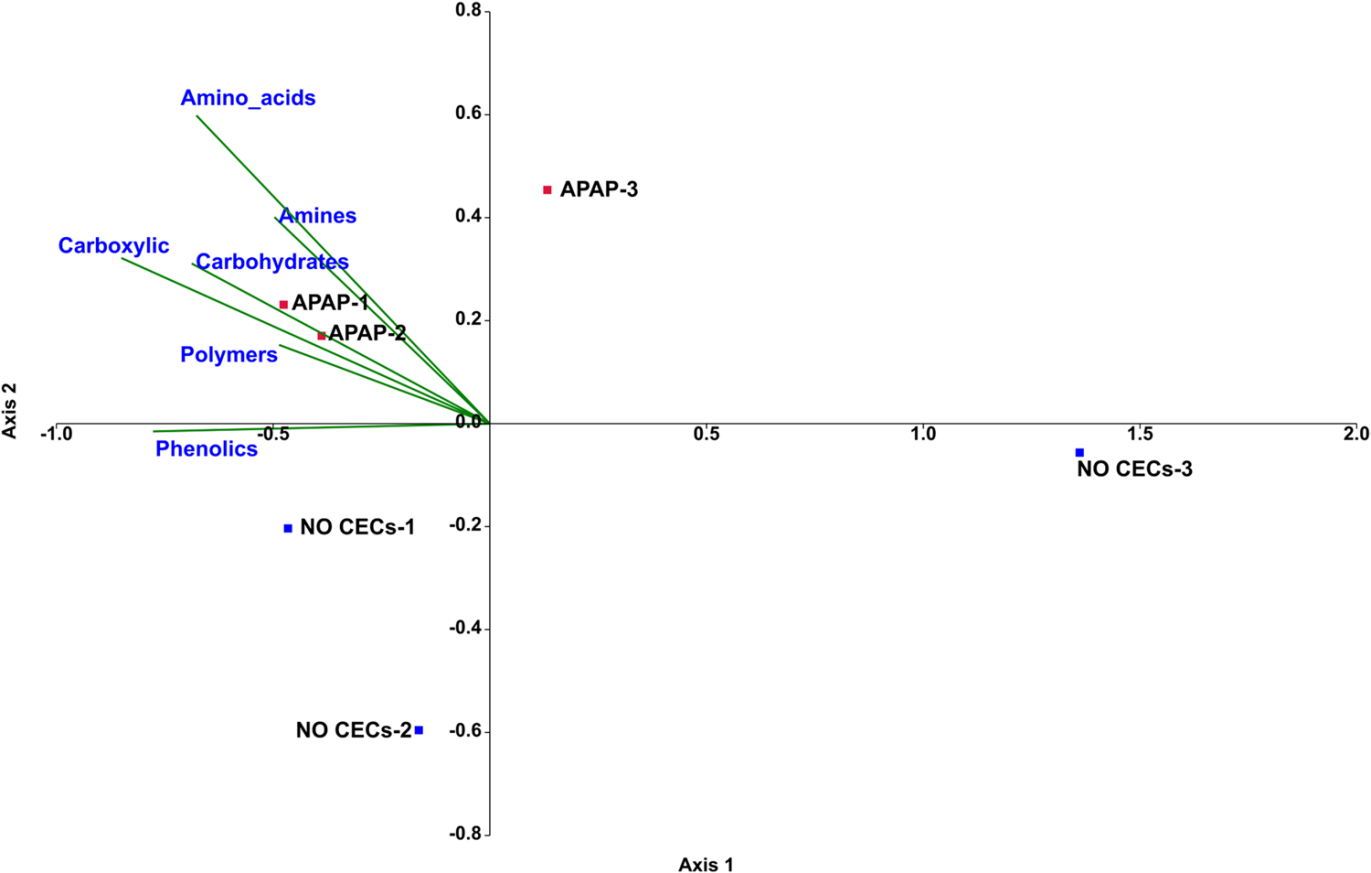
Canonical correspondence analysis in PAST of Biolog Ecoplates that were incubated for 96 h with samples from soil treated with 10 μg/L of acetaminophen and the untreated control and collected 7 weeks (T2) after start of treatment. The figure shows the utilization of the 6 general carbon substrate groups. The vectors, in green, represent a given carbon substrate while vector length indicates the impact of the given factor on community differences. Each treatment had 3 replicates (*n* = 3). APAP-1 = APAP-10 T2 replicate 1, APAP-2 = APAP-10 T2 replicate 2, and APAP-3 = APAP-10 T2 replicate 3

The CCA analyses conducted to determine if substrate utilization could be a factor in shaping soil community differences indicated that amino acid, carbohydrate, carboxylic acids, and polymer metabolism contributed to community structural differences. These had the largest vectors on the CCA plot, indicating that differences in these metabolic pathways between treated and untreated soil communities had a large effect on influencing community structure (Fig. 7).

To confirm the results of the Biolog plates, the FDA hydrolysis assay was used as a proxy for soil community activity. The hydrolysis activities of the different treatments were, 169.94, 161.32, and 154.33 ug of FDA per g of dry soil, for APAP-10, APAP-5, and the untreated soil respectively. The amount of FDA hydrolyzed in the APAP-10-treated soil was significantly higher than in the untreated soil (ANOVA: *F*_2_ = 6.94 P = 0.018; Tukey pairwise comparisons *P* < 0.05). Thus, indicating higher microbial activity in APAP-10-treated soil. These results parallel well with the above Biolog Ecoplate results.

## Discussion

The combined observations of the 16S rRNA data, predicted metagenome, Biolog EcoPlate assays, and FDA analysis indicate that the bacterial communities in our soil samples were sensitive to the APAP concentration used in this study. Significant community differences were observed within 3 weeks of APAP treatment and significant differences in carbon metabolism were observed between treated and untreated samples collected 7 weeks after starting treatment. Taken together, our results show that APAP altered the soil bacterial communities and impacted community functions within a single growing season of eggplants.

In our study, APAP treatment did not change community α-diversity levels as previously observed in another study [69]. This may be due to the fact that plants can stabilize their associated soil microbial communities [42, 70]. Given that microbes are not impacted equally by a disturbance [36, 37], our results suggest that APAP did not completely displace many bacterial taxonomic groups, but caused a shift in the relative abundance of certain groups. Since we observed increases in microbial activity, (i.e., increased substrate utilization and FDA hydrolysis) it is possible that APAP was acting as a carbon source for a subset of soil community members and selected for microbes that can utilize it directly or indirectly [27, 33, 71–73]. The differences in β-diversity between APAP-treated soils and the initial sampling point suggest that the microbial communities were sensitive to APAP at the concentrations found in RWW, especially after 7 weeks of exposure. This is consistent with previous observations that indicated that pharmaceutical products, including APAP, can impact microbial communities, and hinder or disrupt key microbial functions [20–22, 74]. Therefore, our rationale is that since APAP is found in RWW as an intact active compound and is broken down into a glucoside by soil fungi or plants [27, 71, 72] or into the carboxylic acid 2-hexenoic acid by soil microorganisms [33], the intact active compound and breakdown products of APAP can be utilized as carbon sources by soil bacteria [75–77]. Bacterial groups that can utilize these carbon sources will most likely be selected for in APAP-contaminated soils, thus altering the soil microbiome. Plant health is intimately related to its associated soil microbiome and its functions, thus any alterations to the microbiome could have negative impacts on plant productivity [41, 78]

The APAP concentrations used in our study represent levels found in RWW effluent [15, 32, 34, 35, 79]. The exact concentrations of APAP in RWW effluent vary among regions and across seasons, and have been observed to reach concentrations up to 112.78 ug/L, with averages between 0.0081 ug/L [14] and 11.73 ug/L [34, 35]. Additionally, soils that are irrigated with RWW effluent can accumulate between 604 and 4860% of the APAP found in irrigation water [14]. Our results demonstrated that these concentrations can impact soil microbial communities, especially with repeated exposure. Our results concur with previous findings showing that microbes in agricultural soils are sensitive to APAP present in RWW [22, 69, 80]. However, in these other studies, the resolution for detecting specific microbial community members was limited because they relied on non-sequencing-based approaches to characterize changes in the soil microbial community. In contrast, our study was able to detect specific shifts in the microbial community and identify specific bacterial groups that were impacted by APAP treatment using Illumina sequencing-based approaches.

When the microbial community shifts, community functions may also change. Addition of APAP to soils disrupted key aspects of nitrogen cycling although the concentrations of APAP (50 to 1000 mg/L) used in those studies were greater than those found in RWW effluent [22, 74]. Our Biolog assay showed altered microbial functions using concentrations within the range found in RWW effluent (10 ug/L). Besides lower APAP concentration, our study distinguishes itself from previous ones in a few other ways. Unlike previous studies that focused on nitrogen cycling [36, 81], our study examined utilization of 31 ecologically relevant carbon sources (Supplementary Table 1). This approach encompasses a much larger portion of the soil microbial community and was not limited to a specific set of community members such as anammox bacteria [74], or bacteria that contain amoA, napaA, or nifH genes for nitrification, denitrification, or nitrogen fixation respectively [36]. Thus, by using various carbon sources, we screened for a wide variety of bacterial groups that were impacted by APAP treatment. In addition, we employed secondary methods to identify the specific microbes responsible for the observed changes unlike these other studies that examined microbial community functions using Biolog plates [66, 69, 80]. Data obtained from the Biolog Ecoplates does not represent true, in situ, rates for soil bacteria community metabolism, because it only measures metabolism from a subset of organisms capable of growing under laboratory conditions and may not reflect in situ conditions. Despite this shortcoming, many studies have demonstrated that it is a great method to evaluate changes in soil community functions due to disturbances or changes in biotic and abiotic factors [66, 69, 80, 82–84].

In our results, APAP-treated samples had significantly higher rates of carbon utilization in nearly every category measured by 96 hpi (amines, carbohydrates, amino acid, carboxylic acid, and polymer metabolism) compared to the controls. APAP may not be a carbon source for all organisms; therefore, its addition to the soil might have selected for microbes that metabolize it. Liu et al. [66] demonstrated that APAP is broken down in non-sterilized soil, but not in sterilized soil, indicating that soil microbes metabolize APAP. Metabolomics analyses of APAP-treated soil revealed that the microbes break down APAP to 8 identifiable intermediates [66]. The intermediate 2-hexenoic acid, a carboxylic acid, was the most abundant metabolite in the soil after APAP treatment [33]. Therefore, we hypothesized that APAP treatment in our study increased carboxylic acid content in the soil, which in turn led to an increase of microbes that metabolize carboxylic acids. The Biolog assay confirmed our hypothesis to be true by showing a significant increase in carboxylic acid metabolism in APAP-treated soils.

Using PICRUSt2, we developed initial predictions on the expected impacts of APAP on microbial communities. The PICRUSt2 metagenome predictions paralleled the trend of increased carbon metabolism in APAP-treated soil observed in the Biolog assay. The gene prediction data indicated that soil communities treated with APAP may increase in multiple genes for a variety of carbon metabolism pathways, most notably for amino acid and carbohydrate metabolism. This concurred with our Biolog plates data that indicated significantly higher utilization of amino acids and carbohydrates in APAP-treated soils compared to the untreated controls. The CCA of the Biolog assay also indicated that amino acid and carbohydrate metabolism had a strong impact on community shifts between the APAP-10 and untreated soil microbial communities (Fig. 7). Previous studies have also indicated that additional carbon input led to increased soil microbial activity measured as respiration [85–88], FDA dehydrogenase activity [88–91], or microbial biomass [91]. Data from our Biolog assay was congruent with the PICRUSt2 predictions and FDA hydrolysis which all showed increased carbon metabolism after APAP treatment. Therefore, we presume that APAP was acting as a carbon source, and thus stimulating microbial activity. However, additional studies are required to confirm this assumption.

PICRUSt2 predictions were based on functions linked to given 16S rRNA genes that were detected in our soil samples. Thus, shifts in the abundance of 16S rRNA genes may be interpreted as shifts in community functions; however, since these are predictions based on the presence of 16S rRNA genes, these results should be confirmed using another method. By utilizing the Biolog plate assay, we were able to examine changes in metabolic rates for specific substrates, and test these predictions. We observed that shifts in relative abundance of the soil microbial community members were consistent with the observed changes in the microbial community function determined in the Biolog assay. *Amycolatopsis thermoflava* and *Cellvibrio* spp., microbial groups that increased in relative abundance after APAP application, were major contributors to community differences among the soil communities. They are capable of metabolizing a diverse set of carbon substrates, including glycosides [75–77]. Glycosides are major breakdown products of APAP due to microbial activity in the soil [71, 72] and plant detoxification [27]. Their accumulation in plant roots or in the soil probably led to the increase of glycoside metabolizing organisms like *Cellvibrio* bacteria. *Cellvibrio* is a genus of cellulolytic bacteria that are capable of degrading plant cell walls. Some *Cellvibrios* can utilize many different carbohydrates including ⍶- and β-glycosides [75, 77]. These cellulolytic organisms can have major impacts on the soil community by degrading refractory cellulose, and thus making substrates available to other community members [92, 93]. *Cellvibrio* spp*.* can also utilize carboxylic acids, which are another major breakdown product of APAP [33].

Additionally, the relative abundance of Acidobacteria was higher in APAP-treated soils than in untreated controls. This concurs with other studies that observed higher relative abundance of Acidobacteria in the presence of a mixture of pharmaceuticals, including APAP [94]. Examples of Acidobacteria that followed this trend were *Candidatus Koribacter* and *Candidatus Solibacter*. *Candidatus* spp. have optimum growth at pH > 6 [95, 96] and pH plays a significant role in the growth of some Acidobacteria than other factors [96, 97]. The pH of APAP in a saturated aqueous solution is about 6 [98], which may explain why Acidobacteria were more abundant in APAP-treated soils than in the untreated controls. However, not all microbes were tolerant to APAP. For example, there was a decrease in the relative abundance of Actinobacteria in APAP-treated soil compared to the untreated control. Several strains of *Actinomyces* (a genus in the class Actinobacteria) cannot metabolize APAP [71]. This could explain the reduction in the relative abundance of Actinobacteria observed in our study. A group of bacteria identified to the Gemmatiomadetes phylum also decreased in abundance in APAP-treated soil. Two species in this phylum, *Gemmatimonas aurantiaca* and *G. phototrophica* are fastidious with carbon utilization, thus they may not be able to use APAP or its metabolites [99, 100]. Having particular carbon requirements may partially explain the decrease in Gemmatiomonadetes phylum members [101]. However, this needs to be evaluated further.

Irrigation with APAP impacted soil microbes of agricultural importance. For example, *Pseudomonas viridiflava* and *Lysobacter* spp. which increased in soils treated with APAP. *P. viridiflava* is pathogenic to approximately 30 plant species including eggplants, kiwis, tomato, and melon [102]. This pathogen causes soft rot and subsequent browning of the stem or flowering parts, which leads to economic losses to the growers and predisposes the plants to fungal infections [102, 103]. In this study, eggplants were grown in the soil irrigated with APAP, and we think that continued use of irrigation water containing APAP may favor infections by *P. viridiflava. Lysobacter* spp. are recognized for their potential as biological control agents of several plant diseases of economic importance such as *Fusarium* head blight of wheat, brown patch in turfgrass caused by *Rhizoctonia solani*, *Pythium* damping-off of sugarbeet, and summer patch disease of Kentucky bluegrass caused by the root-infecting *Magnaporthe poae* [104–107]. Therefore, irrigation with RWW containing APAP may cause the soils to be suppressive to several fungal diseases.

Our findings highlight the need to investigate the impacts of RWW on plant–microbe interactions. The fact that both plant pathogens and disease suppressive organisms increased in presence of APAP underscores the complexity of soil systems and the impacts of APAP and other CECs found in RWW. The effects of using RWW are multifaceted and many more studies are needed to unravel this issue and to ensure that RWW can be used in a way that continues to persevere clean water supplies while facilitating the growth of healthy crops. Our study demonstrated that APAP concentrations found in RWW can alter soil microbial diversity and functions which may impact plant health and productivity. In spite of the benefits of RWW to agriculture, further investigation into effects of different CECs on soil microbes is needed in order to understand the risk that CECs may pose to natural and agricultural environments.

## Supplementary Information

Below is the link to the electronic supplementary material.

Supplementary file1 (DOCX 25 KB)

## Data Availability

All Illumina sequences will be published on NCBI with accession PRJNA808107.
