# Supplementary material for: Acetaminophen Levels Found in Recycled Wastewater Alter Soil Microbial Community Structure and Functional Diversity
Source: Microb Ecol. 2022 May 4;85(4):1448–62. doi: 10.1007/s00248-022-02022-8 (PMC10167187; doi:10.1007/s00248-022-02022-8)
Supplement: Supplementary file 1 — Supplementary file1 (DOCX 25 KB) [file 248_2022_2022_MOESM1_ESM.docx]

**Acetaminophen levels found in recycled wastewater alter soil microbial community structure and functional diversity**

Nathan K. McLain, Melissa Y. Gomez, and Emma W. Gachomo*

Department of Microbiology and Plant Pathology, University of California, Riverside, Riverside, CA, United States

*Correspondence:

Emma W. Gachomo

emma.gachomo@ucr.edu

**Supplementary Materials and Methods**

Eggplant seedlings of the Patio Baby variety at the 2-4 leaf stage were transplanted into 2 liter pots containing soil collected from organic fields in Bakersfield, California. These are very deep poorly drained soils formed in stream alluvium derived from granitic rock ([Official Series Description - BAKERSFIELD Series (usda.gov)](https://soilseries.sc.egov.usda.gov/OSD_Docs/B/BAKERSFIELD.html#:~:text=The%20Bakersfield%20series%20consists%20of%20very%20deep%2C%20somewhat,artificially%20drained.%20Bakersfield%20soils%20are%20on%20flood%20plains.) [[1]](https://paperpile.com/c/UIqO14/jJ5Fa). The soil did not have a history of irrigation with RWW. The plants for each treatment were maintained on trays and were irrigated 2-3 times a week using a liter of water spiked with either 5 or 10 μg/L of APAP, while the control plants were irrigated with tap water. The treatment started one week after transplanting and was carried out for 10 weeks. Soil cores of 1 cm diameter and 3 cm depth were collected weekly from the rhizosphere of the plants at least 3 cm from the stem and stored at -20 degrees celsius. The treatment was replicated 4 times and each replicate had 5 plants. Soil samples collected before the onset of the treatment (T0), at 3 (T1) and 7 (T2) weeks after starting the treatment were chosen for further analysis. At the T1 and T2 the plants had been irrigated with APAP spiked water 7 and 15 times respectively. Therefore, a cumulative amount 35 μg and 70 μg of APAP had been added to 5 pots of the APAP-5 and APAP-10 treatments respectively at T1, while 75 μg and 150 μg had been added to 5 pots of APAP-5 and APAP-10 respectively at T2.

**Supplementary table 1** All 31 carbon substrates tested by the Biolog Ecoplates.

| Substrate name | Category according to Adams | Category according to Adams | |
| --- | --- | --- | --- |
| **Water (Blank)** | **x** | x |  |
| **B-methyl-D-Glucoside** | **carbohydrate** | carbohydrate | 3 |
| **D-Galactonic Acid y-Lactone** | **carbohydrate** |  | 3 |
| **L-Arginine** | **amino acid** |  | 2 |
| **Pyruvic Acid Methyl Ester** | **carboxylic acid** |  | 4 |
| **D-Xylose** | **carbohydrate** |  | 3 |
| **D-Galacturonic Acid** | **carboxylic acid** |  | 4 |
| **L-Asparagine** | **amino acid** |  | 2 |
| **Tween40** | **polymer** |  | 6 |
| **i-Erythritol** | **carbohydrate** |  | 3 |
| **2-HydroxyBenzoicAcid** | **phenolics** | also a carboxylic acid | 5 |
| **L-Phenylalanine** | **amino acid** |  | 2 |
| **Tween80** | **polymer** |  | 6 |
| **D-Mannitol** | **carbohydrate** |  | 3 |
| **4-HydroxyBenzoicAcid** | **phenolics** | also a carboxylic acid | 5 |
| **L-Serine** | **amino acid** |  | 2 |
| **a-Cyclodextrin** | **polymer** |  | 6 |
| **N-Acetyl D-Glucosamine** | **amine** |  | 1 |
| **y-Hydroxybutyric Acid** | **carboxylic acid** |  | 4 |
| **L-Threonine** | **amino acid** |  | 2 |
| **Glycogen** | **polymer** |  | 6 |
| **D-Glucosaminic Acid** | **carboxylic acid** |  | 4 |
| **ItaconicAcid** | **carboxylic acid** |  | 4 |
| **Glycyl-L-GlutamicAcid** | **carboxylic acid** |  | 4 |
| **D-Cellobiose** | **carbohydrate** |  | 3 |
| **Glucose 1 Phosphate** | **carbohydrate** |  | 3 |
| **a-Ketobutytric Acid** | **carboxylic acid** |  | 4 |
| **Phenylethylamine** | **amine** |  | 1 |
| **a-D-Lactose** | **carbohydrate** |  | 3 |
| **D,L -a-Glycerol Phosphate** | **carbohydrate** |  | 3 |
| **D-Malic Acid** | **carboxylic acid** |  | 4 |
| **Putrescine** | **amine** |  | 1 |

**Supplemental Table 2** The Differentially expressed metabolic genes that were predicted to be upregulated in each treatment after 7 weeks (T2)

| **Venn diagram category** | **KEGG ID** | **Definition** | **General pathway** |
| --- | --- | --- | --- |
| **T2 APAP-H only** | K06001 | tryptophan synthase beta chain | Amino acid metabolism |
|  | K10793 | D-proline reductase | Amino acid metabolism |
|  | K12674 | (carboxyethyl)arginine beta-lactam-synthase | Biosynthesis of other secondary metabolites |
|  | K12675 | Clavaminate synthase | Biosynthesis of other secondary metabolites |
|  | K12673 | N2-(2-carvoxyethyl)arginine synthease | Biosynthesis of other secondary metabolites |
|  | K12676 | Proclavaminate amidinohydrolase | Biosynthesis of other secondary metabolites |
|  | K01452 | Chitin deacetylase | Carbohydrate metabolism |
|  | K19668 | Cellulose 1,4-beta-cellobiosidease | Carbohydrate metabolism |
|  | K07106 | N-acetylmuramic acid 6-phosphate etherase | Carbohydrate metabolism |
|  | K12449 | UDP-apiose/xylose synthase | Carbohydrate metabolism |
|  | K01233 | chitosanase | Carbohydrate metabolism |
|  | K13810 | Transaldolase / glucose-6-phosphate isomerase | Carbohydrate metabolism |
|  | K01179 | endoglucanase | Carbohydrate metabolism |
|  | K15916 | Glucose/manose-6-phosphate isomerase | Carbohydrate metabolism |
|  | K18649 | Inositol -phosphate phostate / L-galactose 1-phospte phosphatase | Carbohydrate metabolism, Amino acid metabolism |
|  | K00844 | Hexokinase | Carbohydrate metabolism, Biosynthesis of other secondary metabolites |
|  | K15052 | propionyl | Energy metabolism |
|  | K00387 | Sulfite oxidase | Energy metabolism |
|  | K12234 | Coenzyme F420-0:L-glutamate ligase | Energy metabolism |
|  | K11212 | 2-phospho-L-lactate transferase | Energy metabolism |
|  | K08256 | phosphatidyl-myo-inositol alpha-mannosyltransferase | Glycan biosynthesis and metabolism |
|  | K06928 | nucleoside-triphosphatase | Metabolism of cofactors and vitamins |
|  | K01492 | phosphoribosylglycinamide | Metabolism of cofactors and vitamins |
|  | K03851 | taurine-pyruvate aminotransferase | Metabolism of other amino acids |
|  | K05553 | minimal PKS acyl carrier protein | Metabolism of terpenoids and polyketides |
|  | K13329 | dTDP-4-dehydro-2,3,6-trideoxy-D-glucose 4-aminotransferase | Metabolism of terpenoids and polyketides |
|  | K05554 | aromatase | Metabolism of terpenoids and polyketides |
|  | K13317 | NDP-4-keto-2,6-dideoxyhexose 3-C-methyltransferase | Metabolism of terpenoids and polyketides |
|  | K05552 | tetracycline polyketide synthase | Metabolism of terpenoids and polyketides |
|  | K14369 | erythromycin 3''-O-methyltransferase | Metabolism of terpenoids and polyketides |
|  | K16435 | dTDP-4-dehydro-6-deoxy-alpha-D-glucopyranose 2,3-dehydratase | Metabolism of terpenoids and polyketides |
|  | K05551 | minimal PKS ketosynthase | Metabolism of terpenoids and polyketides |
|  | K16004 | narbonolide/10-deoxymethynolide desosaminyltransferase | Metabolism of terpenoids and polyketides |
|  | K18445 | diadenosine hexaphosphate hydrolase (ATP-forming) | Nucleotide metabolism |
| **T2 APAP-L only** | K07405 | alpha-amylase | Carbohydrate metabolism |
|  | K11645 | fructose-bisphosphate aldolase, class I | Carbohydrate metabolism, Energy metabolism |
|  | K00002 | alcohol dehydrogenase (NADP+) | Carbohydrate metabolism, Lipid metabolism, Xenobiotics biodegradation and metabolism |
|  | K02805 | dTDP-4-amino-4,6-dideoxygalactose transaminase | Glycan biosynthesis and metabolism |
| **T2 No CECs only** | K00972 | UDP-N-acetylglucosamine/UDP-N-acetylgalactosamine diphosphorylase | Carbohydrate metabolism |
| **APAP-H and APAP-L** | K00693 | glycogen synthase | Carbohydrate metabolism |
|  | K00033 | 6-phosphogluconate dehydrogenase | Carbohydrate metabolism, Metabolism of other amino acids |
|  | K05979 | 2-phosphosulfolactate phosphatase | Energy Metabolism |
|  | K02636 | cytochrome b6-f complex iron-sulfur subunit | Energy Metabolism |
|  | K00956 | sulfate adenylyltransferase subunit 1 | Energy Metabolism, Biosynthesis of other secondary metabolites, Nucleotide metabolism, Metabolism of other amino acids |
|  | K02259 | heme a synthase | Energy Metabolism, Metabolism of cofactors and vitamins |
|  | K18660 | malonyl-CoA/methylmalonyl-CoA synthetase | Lipid metabolism, Amino acid metabolism |
|  | K00632 | acetyl-CoA acyltransferase | Lipid metabolism, Amino acid metabolism, Metabolism of terpenoids and polyketides, Xenobiotics biodegradation and metabolism |
|  | K00949 | thiamine pyrophosphokinase | Metabolism of cofactors and vitamins |
|  | K01674 | carbonic anhydrase | Metabolism of cofactors and vitamins |
|  | K01724 | 4a-hydroxytetrahydrobiopterin dehydratase | Metabolism of cofactors and vitamins |
|  | K02361 | isochorismate synthase | Metabolism of cofactors and vitamins, Metabolism of terpenoids and polyketides |
|  | K16422 | 4-hydroxymandelate oxidase | Metabolism of terpenoids and polyketides, Biosynthesis of vancomycin group antibiotics, Biosynthesis of other secondary metabolites |
| **APAP-L and No CECs** | K00293 | Saccharopine dehydrogenase (NADP+, L-glutamate forming) | Amino acid metabolism |
|  | K14259 | 2-dehydro-3-deoxy-D-arabinonate dehydratase | Carbohydrate metabolism |
|  | K01823 | isopentenyl-diphosphate Delta-isomerase | Metabolism of terpenoids and polyketides |
|  | K00757 | uridine phosphorylase | Nucleotide metabolism, Xenobiotics biodegradation and metabolism |
| **APAP-H, APAP-L, and No CECs** | K16055 | trehalose 6-phosphate synthase/phosphatase | Carbohydrate metabolism |
|  | K01051 | pectinesterase | Carbohydrate metabolism |
|  | K07404 | 6-phosphogluconolactonase | Carbohydrate metabolism |
|  | K18429 | GDP/UDP-N,N'-diacetylbacillosamine 2-epimerase | Carbohydrate metabolism |
|  | K13016 | UDP-N-acetyl-2-amino-2-deoxyglucuronate dehydrogenase | Carbohydrate metabolism |
|  | K18430 | N,N'-diacetyllegionaminate synthase | Carbohydrate metabolism |
|  | K08092 | 3-dehydro-L-gulonate 2-dehydrogenase | Carbohydrate metabolism |
|  | K15862 | cytochrome c oxidase cbb3-type subunit I/II | Energy metabolism |
|  | K01113 | alkaline phosphatase D | Metabolism of cofactors and vitamins |

References

1. [(2006) Bakersfield Series. In: Soilseries.sc.egov.](http://paperpile.com/b/UIqO14/jJ5Fa) <https://soilseries.sc.egov.usda.gov/OSD_Docs/B/BAKERSFIELD.html#:~:text=The%20Bakersfield%20series%20consists%20of%20very%20deep%2C%20somewhat,artificially%20drained.%20Bakersfield%20soils%20are%20on%20flood%20plains.> [Feb 2022](http://paperpile.com/b/UIqO14/jJ5Fa)
